# Overweight and obese adolescents: what turns them off physical activity?

**DOI:** 10.1186/1479-5868-9-53

**Published:** 2012-05-03

**Authors:** Ivana Stankov, Timothy Olds, Margaret Cargo

**Affiliations:** 1 School of Health Sciences, University of South Australia, Adelaide, Australia; 2 Social Epidemiology & Evaluation Research Group, Sansom Institute for Health Research, University of South Australia, Adelaide, Australia; 3 Health and Use of Time (HUT) Group, Sansom Institute for Health Research, University of South Australia, Adelaide, Australia

**Keywords:** Barriers, Physical activity, Adolescence, Obesity, Meta-synthesis, Qualitative synthesis

## Abstract

A systematic review of qualitative studies was undertaken to understand the barriers to physical activity experienced by adolescents who were overweight or obese. From a search of electronic databases and ‘grey’ literature, published between 1950 and 2009, 15 studies met the inclusion criteria. Bronfenbrenner’s model of human development provided an ecological lens for identifying and synthesising barriers to physical activity. Two reviewers appraised study quality. Miles and Huberman’s cross-case analysis was integrated with thematic networking to synthesize the individual, interpersonal and environmental level barriers for boys and girls of different ethnicities and socioeconomic status, across school settings and generalised context. Thirty-five barriers were identified, 13 of which occurred in physical activity situations in the school setting, 18 were not linked to a specific setting, and the remainder were common across both contexts. The fact that these barriers emerged from studies that focused on topics such as victimisation and mental health is particularly poignant and reflects the potentially pervasive influence of adolescent’s excessive weight not only in relation to physical activity situations but other aspects of their lives. Furthermore, socioeconomic status and ethnicity was poorly considered, with only one study linking these participant characteristics to quotations and discussing the potential implications. At present, there are few qualitative studies with sufficiently thick description or interpretive validity that provide insight into this vulnerable group of adolescents, and give them a voice to influence policy and practice.

## Introduction

Physical activity plays an important role in preventing the development of overweight and obesity in young people and stemming its progression into young adulthood. Adolescence is a particularly vulnerable time for the development of obesity because it is marked by a slowing of growth and corresponding decrease in physical activity levels [1]. A significant proportion of adolescents do not meet recommended physical activity guidelines [2,3]. In addition, physically active youth have lower levels of adiposity than youth who are less active [4,5]. Given that there is a much higher risk of overweight adolescents becoming overweight adults [6], engaging young people in physical activity remains a key behavioural target for obesity prevention.

Experiencing obesity during adolescence can have a profound impact on psychosocial development [7] in part because it is a critical period for psychosocial development marked by increasing separation from parents, peer acceptance and identity formation [8]. Adolescents who are overweight are commonly victimised by peers and experience higher rates of low self-esteem, sadness, nervousness and loneliness than peers in the normal weight range [9,10]. Some of these adolescents, in particular girls and younger adolescents suffer depressive symptoms [11] and are more likely to experience suicidal contemplation if subject to weight-based teasing [12]. Heightened body consciousness has been identified as a unique barrier to physical activity for overweight youth as compared to non-overweight youth [13]. Moreover, overweight youth tend to perceive a greater number of barriers to sports participation, including feeling insecure about their appearance [14]. Peer influences are of great importance during adolescence, particularly early adolescence, and may significantly affect the development of attitudes [7] towards exercise and perhaps also programs designed to encourage weight loss. Peer stigmatization can inhibit participation in exercise [15]. Even health educators must take care when assisting these young people as focusing on the obese child’s weight in a negative manner can result in avoidance of exercise programs [16].

Engaging adolescents who are overweight in physical activity requires addressing the barriers that may deter them from participating in the first place. This is important given emerging evidence that these barriers may differ from those of their normal weight peers [14] and persistent evidence that overweight and obese youth are more sedentary and less physically active than their normal weight counterparts [17]. Furthermore, the prevalence of overweight and its cultural acceptance has been linked to ethnicity and socioeconomic status (SES), with greater perceived acceptance evident in those residing in low SES communities/and or with Middle Eastern or Pacific Islander backgrounds [18]. Although current research identifies broadly-defined intrapersonal, interpersonal and environmental barriers to exercise, it is unclear how these barriers are experienced by and deter overweight and obese adolescents from engaging in physical activity in those settings responsible for their socialisation. Such information is needed to develop effective engagement strategies for this group of vulnerable adolescents.

### Aims and objectives

This study aims to strengthen the evidence on understanding the barriers to physical activity experienced by overweight and obese adolescents. Study objectives aim to:

1. synthesize evidence from qualitative research reporting on the barriers to physical activity experienced by adolescents who are overweight or obese with attention to socioeconomic and ethnic differences; and

2. provide gender, socioeconomic, ethnic and setting-specific implications for engaging adolescents who are overweight and obese in physical activity.

Qualitative synthesis is appropriate for consolidating evidence across studies to develop new explanations for a body of research [19,20]. A key strength of qualitative research is the insight that it provides into the perceptions and attitudes of those experiencing a particular phenomenon of interest [21]. Consequently, Miles and Huberman’s cross-case analysis [22] was integrated with thematic networking [23] to understand the barriers experienced by overweight and obese adolescents to engaging in physical activity or exercise at school, home and in the community.

## Methods

### Selection criteria

Given the interest in obtaining primary studies detailing overweight and obese adolescents’ experiences of engagement in physical activity, all studies were screened against these inclusion criteria:

1. presented data using qualitative individual or group interviews;

2. included participants 10–20 years old, with an emphasis on adolescent participants i.e. 12–18 years old, or adults reflecting back on this adolescent timeframe;

3. participants are/were overweight, obese or morbidly obese during adolescence and had no intellectual or physical disabilities; and

4. explored participants’ experiences of physical activity and barriers to exercise participation

OR in the case of a general population studies which stratified/separated results for overweight or obese youth.

OR studies which addressed adolescent overweight/obesity and generated findings which include barriers to physical activity.

### Search strategy

The search for relevant studies was conducted using terms in Table 1 and was limited to articles written in English. ‘Physical activ*’ and ‘exercise*’ as well as ‘barrier*’ were excluded as search terms for two reasons. First, preliminary searches including these terms narrowed the results significantly and few relevant articles were retrieved. Second, a search without these terms revealed that ‘physical activity’ and ‘barrier’ were not always present in the keywords, abstract or title of relevant publications, instead emerging as key findings in qualitative studies on related topics (e.g. stigmatisation). The search strategy ensured that such studies were included, especially given the limited number of qualitative studies addressing barriers to participation.

**Table 1:** **Qualitative assessment criteria utilised in the Critical Appraisal Skills Programme (CASP) for qualitative methodologies**[24]

Screening questions	Clear statement of research aims Appropriateness of qualitative methodology
Research design	Justification for research design
Sampling	Explanation and justification for recruitment of participants
Data collection	Transparency and justification in relation to data collection methods
Reflexivity	Specification of relationship between researcher and participants
Ethics	Evidence of informed consent and ethical approval
Data Analysis	Explanation and evidence of rigor in the process of data analysis
Findings	Explicitness of findings and consideration to credibility of findings
Value of research	Contribution to knowledge and transferability of findings

**Figure 1:**
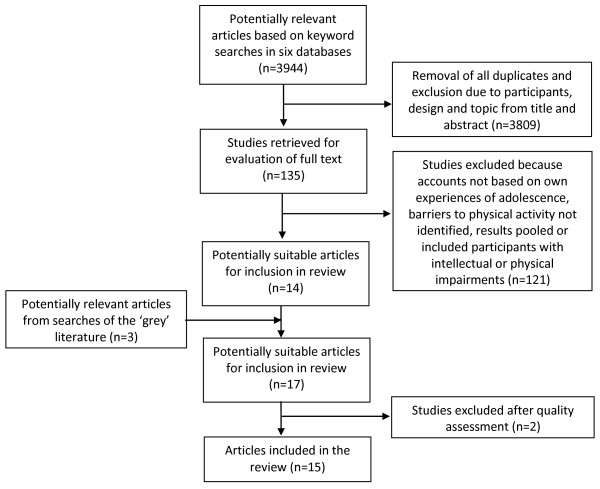
Flow diagram illustrating studies meeting inclusion criteria.

A comprehensive search for qualitative studies published between 1950 and the end of 2009 in Medline, Embase, CINAHL, Psychinfo, SportsDiscus and Academic Search Premier retrieved 3944 studies (Figure 1). Article titles and where appropriate, abstracts were screened, irrelevant studies and duplicates excluded. The 135 remaining studies were retrieved and the full text examined by two independent assessors; 14 studies met the inclusion criteria. Three studies were identified through searches of non-peer reviewed ‘grey’ literature (i.e. Google Scholar, World Wide Web and professional networks) and pearling reference lists (Figure 1); however only one of these met the inclusion criteria.

### Quality assessment

Two assessors independently rated study quality using the Critical Appraisal Skills Programme (CASP) for qualitative methodologies [24]. A summary of appraisal items appear in Table 2. Consensus between assessors was achieved through discussion. Studies of poor quality were excluded based on two screening questions.

**Table 2:** Descriptive characteristics of the 15 studies included in the qualitative synthesis

**Study number/Author/Country/Recruitment setting**	**Aims and fit to the systematic review**	**Design/method of data collection**	**Population characteristics**
1. Lee et al. (2009) [25], Taiwan, primary schools in Taipei City	·To explore viewpoints on exercise and reasons for not exercising among obese preadolescents in the precontemplation stage	Not explicit, semi-structured focus group discussions	N = 11, Boys & girls, aged 11–13 years, BMI – not specificed (obese) according to Taiwan’s Department of Health Executive Yuan (2002)
	·To identify key motivators for encouraging overweight or obese children to engage in, or increase regular exercise		
	**Fit:** focus on reasons for non participation in exercise		
2. Thomas & Irwin (2009) [26], Canada, physician’s office, cross-cultural learners’ centre, YMCA, girl guides leader, neighbour, mother	·To assess overweight and obese adolescents’ perceptions of the meaning of “healthy body weight”, facilitators and barriers to healthy body weight attainment, and program components they believed would effectively enhance and support health body weight behaviours.	Not explicit, semi-structured one-on-one in-depth interviews	N = 11, Boys & girls, aged 14–16 years, overweight (BMI > 85^th^ percentile) or obese by self report (BMI > 95^th^ percentile according to the Centres for Disease Control and Prevention (2000)
	**Fit:** from the perspective of barriers to “healthy body weight” attainment		
3. Trout & Graber (2009) [27], United States, weight loss camp from the Midwestern US	·To explore the overweight students’ experiences in and perceptions of physical education	Not explicit, one-on-one open-ended interviews and follow-up telephone interviews	N = 12, Boys & girls, aged 13–18 years, BMI > 85^th^ percentile (overweight) according to the Centres for Disease Control and Prevention
	**Fit:** experiences of physical education classes		
4. Griffiths & Page (2008) [28], United Kingdom, Care of Children with Obesity Clinic, Royal Hospital for Children	·To extend our understanding of weight-relation victimisation experiences of obese young people and how, in particular, this impacts on peer relationships **Fit**: from the perspective of victimisation and peer relationships	Interpretive Phenomenological Analysis, semi-structured in-depth interviews	N = 5, Girls, aged 12–18 years, BMI > 95^th^ percentile (obese) according to Cole, Freeman & Preece (1995)
	**Fit**: from the perspective of victimisation and peer relationships		
5. Daley et al. (2008) [29], United Kingdom, community & children’s hospital for evaluation of obesity	·To explore obese adolescents’ experiences of participation in an exercise intervention	Not explicit, semi-structured interviews	N = 25, Boys & girls, aged 11–16 years, BMI > 98^th^ percentile (obese) according to Cole, Freeman & Preece (1995)
	**Fit**: qualitative study nested within a RCT focusing on experiences of intervention		
6. Curtis (2008) [30], United Kingdom, community-based obesity intervention programme	·To explore the experiences of young people with obesity within the secondary school environment in relation to areas of concern prioritised by the Health School Program	Not explicit, focus groups & one semi-structured interview	N = 18, Boys & girls, aged 10–17 years, BMI – not specified (obese)
	**Fit**: experiences of secondary school generally		
7. Boyington et al. (2008) [31], United States, hospital based paediatric diabetes screening & prevention program	·To explore cultural attitudes & perceptions toward body image, food, and physical activity among a sample of overweight African American girls	Not explicit, semi-structured group interviews	N = 12, Girls, aged 12–18 years, BMI – not specified (screened as overweight)
	**Fit**: from the perspective of general cultural attitudes relating to body image, food & exercise		
8. Alm et al. (2008) [32], United States, subset of participants involved in Teenways pilot project	·To explore the reasons for weight management ·To identify barriers & facilitators of reaching behaviour goals	Not explicit, semi-structured interviews	N = 18, Boys & girls, 13–16 years, BMI > 95^th^ percentile (obese) – which index not specified.
	·To identify barriers & facilitators of reaching behaviour goals		
	·To investigate the role of a motivational behaviour coach in goal-setting among obese Bronx adolescents in a weight management program		
	**Fit**: from the perspective of achieving behaviour goals in a weight management program		
9. Bodiba et al. (2008) [33], South Africa, the University of Limpopo – Turfloop Campus	·To explore adolescents’ attitudes, feelings and needs regarding their body mass	Not explicit, semi-structured focus group interviews	N = 75, Girls, 17–19 years, no BMI criteria but BMI > 25 kg/m^2^ classed as overweight according to Senekal (1988)
	·To identify social limitations encountered by female adolescents as a result of their body mass		
	·To investigate feelings in relation to the societal emphasis on weight loss		
	·To explore relationship between body mass & self-concept		
	·To identify whether there are differences in self-concept between female adolescents with low, average and high BMI		
	**Fit**: from the perspective of self concept in relation to social context		
10. Langley (2006) [34], United States, wellness program run at local recreation centre	·To understand the factors influencing PA participation for middle school girls who are overweight or at-risk for overweight	Not explicit, focus groups & reflective journals	N = 17, Girls, aged 11–13 years, BMI > 85^th^ percentile (overweight) according to CDC (2000)
	·To examine the effects of a recreation centre’s wellness program on PA levels and the determinants of PA participation in middle school girls who are overweight		
	**Fit**: focus on physical activity barriers by one research question		
11. Wills et al. (2006) [35], United Kingdom, schools and youth groups in areas within Eastern Scotland	·To discover whether, & how, weight and body size infiltrate other areas of teenagers’ everyday lives	Not explicit, semi-structured interviews	N = 36, Boys & girls, 13–14 years, Normal weight to obese (BMI > 30) according to Cole et al. (2000)
	·To discover how these issues are experienced & perceived		
	·To discover whether medical definitions of fatness are reflected in young peoples’ discursive concerns		
	**Fit:** from the perspective of body image perceptions among teenagers, stratified by weight status		
12. Smith (2000) [36], United States, National Association to Advance Fat Acceptance (NAAFA) and community	·To learn about adults’ experiences as obese adolescents	Grounded Theory and Symbolic Interactionism, semi-structured interviews	N = 24, Boys & girls, aged 25–40 years reflecting back on their experiences as obese adolescents
	·To review literature related to the areas of childhood obesity, physical attractiveness, discrimination, and stigma		
	**Fit:** from the perspective of weight salience		
13. Neumark-Sztainer et al. (1999) [37], United States, urban public high school	·To gather descriptions of experiences related to body & self-image from overweight adolescent girls to understand how they view themselves & relate to their social context	Not explicit, semi- structured interviews	N = 50, Girls, aged 14–20 years, BMI > 85^th^ percentile (overweight) according to Himes & Dietz (1994)
	·To compare experiences related to self-image/body image, among African-American & Caucasian overweight girls		
	**Fit**: from perspective of self-image		
14. Neumark-Sztainer, Story & Faibisch (1998) [10], United States, urban high schools	·To explore how African-American and Caucasian adolescent girls describe weight-related stigmatisation experiences and their response to these experiences	Not explicit, semi-structured interviews	N = 50, Girls, aged 15–17 years, BMI > 85^th^ percentile (overweight) according to Himes & Dietz (1994)
	**Fit**: from the perspective of stigmatisation		
15. Smith & Perkins (2008) [38], United States, recently completed nutrition & exercise program supported by the American Academy of Pediatrics	·To explicate the meaning of being overweight for adolescents attending a medical clinic for weight reduction	Phenomenology, story path/conversation	N = 3, Boys & girls, aged 16–18 years, 41 < BMI < 49
	**Fit**: from the perspective of mental health		

*BMI* body mass index, *RCT* randomised controlled trial, *PA* physical activity.

### Data analysis

Bronfenbrenner’s model of human development [39] provided an ecological lens for synthesising the barriers to physical activity experienced by adolescents who were overweight or obese and translating study findings into implications for engaging adolescents into physical activity. The Progress-Plus Framework [40] was used to further synthesise barriers by participant ethnicity and SES. Given the variation apparent in the primary studies, the extent to which each was considered was represented by the following three categories; 0 = participants’ ethnicity and/or SES not addressed in the primary study, 1 = descriptive information on the ethnicity and/or SES of participants presented in a table or summarised descriptively in the text, 2 = the ethnicity or SES of one or more participants is linked to at least one quote denoting a barrier, and 3 = ethnicity and/or SES are synthesised and discussed.

Miles and Huberman’s cross-case analysis was integrated with thematic network analysis [23] to synthesize the individual, interpersonal and environmental level barriers for boys and girls across school, community and home settings and across different ethnic and socioeconomic backgrounds. The application of thematic network analysis [23] follows Pocock et al. [41], and was used to identify and ascribe interpretations to the barriers experienced by adolescents in relation to engagement in physical activity. ‘Basic themes’ represent the lowest order premises and were grounded in participant experience, as detailed in each primary study. These codes were compared and contrasted for similarities and differences and then grouped into more abstract ‘organising themes’ guided by the reviewer’s interpretations of the primary findings. Higher-order ‘global themes’ encompassed the principal metaphors across the studies included in the review. The categories of individual, interpersonal and environmental from Bronfenbrenner’s ecological model [39] of human development were used as the deductive ‘global themes’ which guided the inductive synthesis and identification of ‘organising themes’ or barriers to physical activity. Through the application of Miles and Huberman’s principles of cross-case analysis, conceptually-ordered displays were constructed. For example, based on participant experiences the basic theme of ‘physical bullying’ was assigned to matrices for both genders in the school setting under the higher-order deductive theme ‘interpersonal’. The coding was consolidated as the organising theme of ‘victimisation’ was abstracted to give meaning to adolescents’ experiences of ‘social exclusion’, ‘stereotyping’, ‘verbal bullying’ and ‘physical bullying’. Thus, inductive organising and basic themes were located under deductive global themes from Bronfenbrenner’s model. Two reviewers independently reviewed and discussed the coding for each primary study; the findings reflect the consensus achieved.

## Results

### Quality assessment

Application of the CASP appraisal tool [24] revealed considerable variation in study quality. Two studies were excluded [42,43] due to inadequate methodological explanation. Only three of 15 studies explicitly identified the qualitative approach used. Phenomenology was used in two studies and grounded theory in a third study. Most studies (67%) conducted individual interviews, while four used focus groups and only one study used both methods. Generally, studies provided little detail on data analysis, researcher reflexivity and strategies used to ensure rigour. Sampling was well reported by all studies, with one exception [42] where inconsistencies and errors in reporting were apparent.

### Description of the studies

The 15 studies in the review were published between 1998 and 2009 (Table 2). Of these, eight were from the United States, four from the United Kingdom and one from South Africa, Canada and Taiwan. Only four studies [25-27,34] focused on understanding barriers to physical activity experienced by adolescents, as one of their main research questions. Remaining studies addressed related issues such as victimisation [28], self-concept [33] and cultural attitudes [31] with barriers to physical activity emerging as themes within their findings, as indicated in Table 2. Nine studies included both genders while the other six studies included girls only. Although all studies targeted participants who were overweight or obese during adolescence, only nine studies reported on the body mass index (BMI) criteria used to classify overweight or obesity.

Furthermore, ethnicity and SES were considered to different extents in the primary studies, as evident in Table 3. Eight of 15 studies related ethnicity and/or SES to at least one barrier via link to quotation in the results section [29,34], synthesising the potential influence of ethnicity and/or SES in the discussion section [10,31,33,35,36] or both [37].

**Table 3:** Level of consideration of socioeconomic status (SES) and ethnicity in primary studies

**Study**	**Country of study**	**SES**	**Ethnicity**
1. Lee 2009	Taiwan	0	1*
2. Thomas & Irwin 2009	Canada	1	1*
3. Trout & Graber 2009	United States	1	0
4. Griffiths & Page 2008	United Kingdom	0	0
5. Daley 2008	United Kingdom	2	2
6. Curtis 2008	United Kingdom	1*	0
7. Boyington 2008	United States	1	3*
8. Alm 2008	United States	1*	1
9. Bodiba 2008	South Africa	0	3*
10. Langley 2006	United States	0	2
11. Wills 2006	United Kingdom	3*	0
12. Smith 2000	United States	3	3
13. Neumark-Sztainer et al. 1999	United States	0	2 & 3
14. Neumark-Sztainer, Story & Faibish 1998	United States	1*	3
15. Smith & Perkins 2008	United States	0	0

0 Not addressed – no attention paid to ethnicity &/or SES.

1 Descriptive –descriptive information was provided on the ethnicity &/or SES of participants.

2 Linked to quote – link was made between the ethnicity &/or SES of a participant and their corresponding quote.

3 Synthesised findings – attempt made to synthesise and discuss the influence of ethnicity &/or SES in the discussion.

* All participants from the same SES or ethnic group as indicated by the column in which it is displayed.

### Thematic findings

Thirty five basic themes were identified under 14 higher-order organising themes. Ten of these basic barrier themes operated at the environmental level, six at the interpersonal level and a further 19 at the individual level. Barriers identified in the school setting and generalised context (including community and home) were grouped according to the initial deductive global themes (environmental, interpersonal and individual) informed by Bronfenbrenner’s model [39].

**Figure 2:**
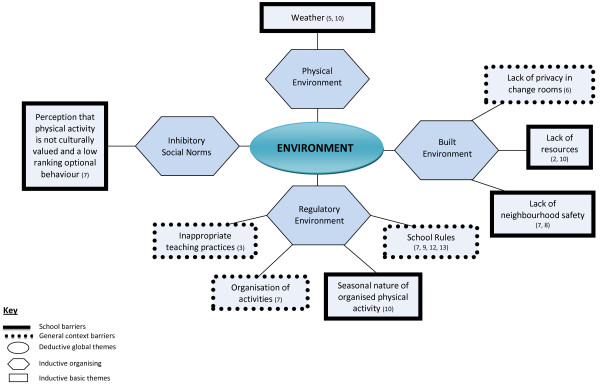
Thematic network depicting environmental barriers.

**Figure 3:**
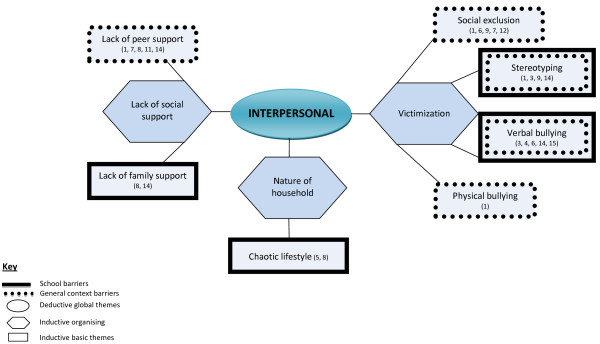
Thematic network depicting interpersonal barriers.

**Figure 4:**
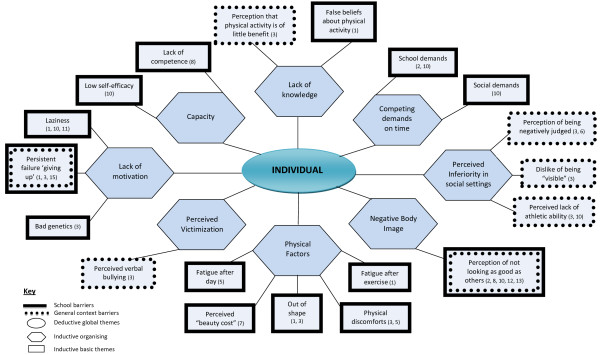
Thematic network depicting individual barriers.

Findings are displayed graphically in Figures 2, 3, 4 with global themes appearing in circles, organising themes in hexagons and basic themes in boxes. Given overlap in the themes by setting, the barriers are described under the relevant global themes with school-related barriers appearing in dotted boxes, barriers in the general context appearing in solid boxes and those identified in both settings depicted in a dotted box nested within a solid box. To summarise the barriers by setting, at the environmental level, five basic barriers operated in the general setting and the remaining five in the school context. At the interpersonal level however, three barriers were experienced in the school, one in the general context and the remaining two in both settings. Furthermore, at the individual level, five basic barrier themes emerged in the school setting, 12 in the general context and two in both domains. Basic themes are underlined to facilitate their identification in Figures 2, 3, 4.

No gender differences were identified in studies including both boys and girls. Given that six studies only included girls, barriers identified as ‘unique’ to girls in these, were discussed as such in relevant sections. Similarly, barriers identified in studies including participants from the same ethnic and/or SES group or studies which reported unique barriers for specific ethnic or SES groups in quotations, were discussed in relevant sections.

Quotations from primary studies which represent basic themes in thematic network coding are presented to enhance the interpretive validity of the synthesis. The quotations that best represented each theme were selected, where numerous quotations of the same theme were available, across different studies.

### Environment

#### Regulatory environment

Within the regulatory environment of the school, two school rules, the organisation of activities as well as inappropriate teaching practices acted as barriers to engagement. The first rule related to adolescents having to wear a uniform or follow a dress code during physical education (PE) classes. This often left them feeling uncomfortable due to body image concerns where too much of their body was revealed [36]. Swimming was one situation that was identified. Attempts at trying to avoid such situations by wearing a t-shirt, for example, were refused by teachers:

“And I don’t want to wear a swimsuit. I asked him [teacher] nicely, ‘please do I have to wear a swimsuit?’ ‘Yes, you got to wear a swimsuit…’ And the only swimsuit I got is one that shows my back and I don’t want it to show my rolls-that’s gross. And then people going to call me Free Willy or something.” ([37], p.316)

The second rule related to the inability of adolescents to meet certain qualifying prerequisites for blood pressure and/or weight which created a further barrier to participation in certain sports, for girls. Furthermore, the way in which activities were organised, including high enrolment rates was identified in Boyington et al. [31] as limiting African American girls’ engagement in physical activity as interest in certain activities was higher than what could be realistically accommodated. Moreover, the fact that desired activities were not offered by the school also impeded girls’ efforts to be physically active. Inappropriate teaching practices also deterred adolescents from participation, especially when teachers punished the whole class when an overweight adolescent “slowed to a walking pace” [27].

Within the generalised context, the seasonal nature of organised physical activity was described by girls as promoting sedentary behaviours during times of the year where their desired activities were not offered. As a result it was a barrier to continued “year-round” engagement in physical activity ([34], p.52).

#### Built environment

The lack of privacy in change rooms at school, in particular, hindered engagement during PE lessons. Although facilities were gender segregated, there were places where the overweight body was put on “display”, which made youth feel uncomfortable:

“…the worst bit was getting changed and getting into the uniform for PE, which was shorts.” ([30], p.413)

A lack of resources was an additional barrier for adolescent girls. The reason that girls did not engage in seasonal organised physical activities was due to a lack of facilities [26], transportation or money [34]. Living in small rural communities put additional constraints on engaging in physical activity as selection was limited: “There really isn’t much to do here” ([34], p.53).

Within the built environment in the generalized context**,** girls identified the lack of neighbourhood safety as a major reason for non-participation in physical activity [31,32]. In these instances girls lived in the inner-city or were from a predominantly low income sample.

#### Inhibitory social norms

An inhibitory cultural norm surfaced as influencing participation in physical activity in one study with African American participants. The perception that physical activity is not culturally valued and is a low ranking optional behaviour was described by girls. Boyington et al. ([31], p.5) stated that some adopted the attitude of “I’ll do it [physical activity] later” because in addition to being a low ranking behaviour in African American culture, physical activity was identified as taking up too much time and resources.

#### Physical environment

Factors associated to the climate, including weather conditions were identified by two studies as inhibiting engagement in physical activity [29,34].

### Interpersonal

#### Victimization

Adolescents in these studies experienced four types of victimization: verbal and physical bullying, social exclusion and stereotyping. Verbal bullying and stereotyping were experienced in both contexts while social exclusion and physical bullying were featured in the school setting only.

Adolescents carrying extra weight were verbally bullied by their peers during school PE lessons. This bullying was triggered by engagement in strenuous activities which “emphasise the overweight child’s body”, for example trampolining:

“I get bullied, you know at school, at…and I get bullied at PE because when…we had trampolining and I didn’t want to go in then, because people would like take the mickey.” ([30], p.413)

Unfortunately, PE classes are mandatory unless adolescents have a note to be excused from class. This leaves them in an inescapable situation of having their body on display and vulnerable to the influence of insensitive peers. Reports of name calling, such as “Shamu [the whale]” ([27], p.279) during swimming lessons were common. There is further indication that verbal bullying is persistent, raised here in relation to the generalised context:

“In the gym, they laugh and talk behind my back,” and “It hurts me when they say ‘hey there, fat kid.’ I try to ignore them, but it does not stop.” ([38], p.392)

Taiwanese adolescents also expressed being physically bullied due to their overweight in school PE classes:

“I don’t like it because I’m fat and often get hit by others. When we play dodge ball, I’m often the target that gets hit, so I don’t like it.” ([25], p.173)

In addition to overt acts of verbal and physical bullying, adolescents were socially excluded by peers at school [36] and were pushed to the “outside of local peer culture”:

“you were classed, if like in the first year, you were decided that you were one of the outcasts, you weren’t the same as everybody else you were pushed to the outside and you weren’t let in …” ([30], p.414)

Social exclusion often occurred below the radar of teachers and left adolescents on their own to cope with this taunting.

Stereotyping was identified as affecting girls’ involvement in physical activity in both the school and general contexts. This was discussed in relation to negative stereotypes that others hold of individuals who are overweight, for example that they are inactive or lazy. Furthermore, adolescents who are overweight were also perceived to be “unable to do certain physical activities” [10] like dancing for example, and in some cases were even stereotyped by teachers who would say “It’s a little too strenuous… you might want to sit this one out” ([27], p.278). Such victimization from figures of authority was particularly frustrating as in this case, the adolescent wanted to participate.

#### Nature of household

Adolescents living in noisy and cramped households led a “chaotic and unstructured lifestyle” with friends and family members frequently hanging out at their home and having parents who worked long hours or multiple jobs ([32], p.281). The nature of such chaotic households was described as a barrier to engagement in structured physical activity. The lack of predictability of household events made it challenging for young teens (15 years) to attend activities on time, especially if they required assistance with transportation.

#### Lack of social support

The lack of peer support took on different forms. It was identified as a blatant disapproval from peers who were opposed to the adolescents’ weight loss attempts, which included physical activity. Alm et al. described those adolescents who had a significant other who preferred a “voluptuous” ([32], p.281) partner and those with friends who were overweight and opposed weight loss encountered significant barriers to engagement. Additionally, Wills et al. identified that not having friends to exercise with hindered participation [35] as exercising alone made adolescents “…bored and [that the exercise] has no purpose at all.” ([25], p.174)

Lack of family support was identified by overweight adolescents from low SES backgrounds as affecting engagement. Parental discouragement through blaming for their overweight and sedentary lifestyle was described as a barrier to behaviour change in adolescents attending a weight management program which included physical activity [32]. Furthermore, parents who were not trying to improve their own activity levels hindered engagement because adolescents often did not have anyone to be active with.

### Individual

#### Negative body image

The negative perception and awareness of one’s own body in relation to physical appearance was experienced as a barrier to physical activity at school. What emerged as salient was the perception of not looking as good as others during PE lessons. Such perceptions related to the adolescents’ views of how they looked in clothing worn during exercise. One study reported that these perceptions at school led to feelings of embarrassment in girls, particularly whilst in front of boys:

“I hate gym class. I hate wearing shorts. I feel so embarrassed about how I look in shorts.” ([32], p.281)

Such perceptions caused embarrassment in the generalized context and adolescents expressed feeling “uncomfortable” ([26], p.113), and led to the avoidance of certain activities:

“I am embarrassed of the way I look in a bikini, but everyone wears bikinis so I don’t swim anymore.” ([34], p. 52)

Adolescents internalised the experiences of having their bodies on display in unavoidable physical activity situations and became self-conscious, embarrassed and even fearful of becoming a target of further victimization.

#### Perceived victimization

The perception of being verbally bullied by peers during PE classes appeared to stem from the fact that adolescents felt exposed in having to be active in front of others. Some adolescents who were rarely teased expressed feeling as though peers were bullying them “behind my back” ([27], p.280). During competitive situations such perceptions were heightened, for instance during exercise testing:

“You’d be the first one out (on the Pacer test) and everyone would look down on you and be like, “huh, they can’t do it; they’re overweight.” ([27], p.281)

#### Perceived inferiority in social settings

The perception of low rank or importance compared to others in the social context was multi-faceted and created significant barriers to engagement.

Adolescents’ perceptions of being negatively judged by others during PE lessons negatively impacted engagement as identified by two studies:

“Everyone stares at you, you become the target when it’s PE, even more so, even if you’re not scared because you think that you’re going to become the target, and you know that you can’t do that area or whatever and you become more self-conscious at which point you get bullied more.” ([30], p.413)

Such perceptions were highly important to adolescents, with most discussions of PE involved references to peer comparisons.

Perceptions of reduced athletic ability relative to others also deterred engagement, particularly in overweight adolescent girls. This was discussed as causing embarrassment during PE lessons at school:

“I’m not very good at a lot of things we do in PE so I get embarrassed when we do those things.” ([34], p.52)

Boys reported similar experiences during PE day, expressing their lack of success in various events:

“When we’d have PE day at school and your whole school goes out to PE, I never won anything. I never won the kickball contest. I never won the basketball shooting contest, I never won the Frisbee throw.” ([27], p.277)

Furthermore, a dislike of being ‘visible’ was expressed by adolescents in the study by Trout and Graber [27]. Adolescents expressed their dislike of PE classes at school because they transpired in a public arena where their overweight body and lack of success was on display to others. Often the dislike of being “visible” ([27], p.279) was far worse than that of the activity.

#### Competing demands on time

Adolescents have a number of competing interests, obligations and commitments. Social hobbies such as reading and surfing the internet as well as obligations to family and friends limited opportunities for engagement as described by African American girls [34]. Furthermore, school and homework commitments as well as lengthy travel to and from school for adolescents living in rural communities significantly reduced the time available for adolescents to engage in physical activity [26].

#### Lack of motivation

Youth made attributions about their lack of motivation. In the general context lack of motivation was attributed to laziness by a number of studies. Langley reports that the adolescent girls felt “too lazy” ([34], p.52) and that they “can’t be bothered” ([35], p.400) and “…don’t want to move, don’t want to go out” ([25], p.173). In the study by Trout and Graber [27] adolescents identified inheriting “bad genetics” which caused them to fail during PE classes.

Persistent failure during physical activity was featured in both school and general contexts in a number of studies. Adolescents, who struggled to lose weight through exercise and did not achieve physical rewards, often gave up:

“I really try and sometimes give up, but most of the time I watch. It is very hard” and “It seems like I’ve tried everything, watching my diet and exercising, and nothing works.” ([38], p.392)

The failure experienced was directly attributed to being overweight and resulted in a lack of motivation to be physically active:

“I think the weight caused [failure in PE]. Because I was overweight I didn’t want to make an effort. I didn’t want to try because I knew I wouldn’t be good at it.” ([27], p.283)

#### Physical factors

Various physical factors have been attributed to hindering participation of adolescents. Among the most prominent of these are physical discomforts including “dying of breath” ([27], p.280,[29]); knee and joint pain and sharp pains in the chest, all of which were attributed to being overweight:

“…you knew it was all because you were overweight. I hated going to PE.” ([27], p.280)

Being too out of shape was also heavily featured as a barrier to engagement. Three studies featured this barrier, emphasising the difficulties in being active as an overweight individual:

“…I like to ride a bicycle, but I can only do so for a short period of time because I feel very, very tired, and I have no strength.” ([25], p.174)

Furthermore, fatigue after the day[29] was described as a barrier to participation in physical activity in the general context by a Caucasian adolescent. Similarly, the anticipated fatigue after exercise was described as a barrier by Taiwanese adolescents who felt they could not do anything after they finished exercising:

“…if I exercise, I have to exercise for a long time to burn calories, and I think this will make me very tired so that I cannot do other things.” ([25], p.173)

Finally, the perceived “beauty cost” or undesirable impact on appearance as a result of physical activity emerged as a barrier in African-American girls an appeared to outweigh the health benefits. This was described through externalised comments and the reference to “others”:

“[Girls are] not interested in working like real hard doing all that exercise stuff. Some of them like – like girls they don’t like to sweat and get their hair messed up…They think like they do the wrong thing, they break their nails, it’s a crisis.” ([31], p.5)

#### Capacity

The lack of competence in setting goals was described by Alm et al. [32] as limiting participation. Adolescent girls from low SES backgrounds described setting unrealistic goals and pursuing these in an “all-or-nothing manner” or wanting to exercise but with no specific activities in mind. Low self-efficacy or low confidence in the ability to seek support given problems with transportation or other competing activities was a barrier to continued participation in exercise in Caucasian girls [34]. The adolescents who described transportation issues for example, did not pursue alternative methods including “getting a ride with a friend or car pooling” ([34], p.53).

#### Lack of knowledge

One study reported on instances where Taiwanese adolescents held false beliefs about physical activity, namely the fact that playing computer games was comparable to exercising:

“I spend so much time on the computer that I think I’ve already burned up a lot of calories and am doing exercise.” ([25], p.174)

The perception that physical activity is of little benefit was also a belief which impeded participation in PE at school:

“I gained weight gradually every year, like five or ten pounds. PE didn’t do anything for me.” ([27], p. 277)

## Discussion

A synthesis of 15 qualitative studies points to four key findings worthy of discussion. First, adolescents who are overweight and obese experience a range of barriers to participation in physical activity; some barriers are specific to this group while others potentially apply to all adolescents. Others, such as physical discomfort and fatigue, while general in nature, are exacerbated by the weight status of the adolescent. Second, and relatedly, environmental and interpersonal circumstances may reinforce negative self-perceptions in overweight and obese adolescents in school or generalized settings at a critical point in their psychosocial development, thus magnifying their psychosocial vulnerability. Third, it would appear that girls may confront barriers to physical activity not shared by boys. Forth, there is some indication that barriers may be uniquely experienced by adolescents from low SES backgrounds and certain ethnicities. The findings point to implications for engagement of this vulnerable group of adolescents in physical activity.

### Nature of barriers

Evidence from previous studies shows that environmental level barriers such as lack of resources [44], lack of neighbourhood safety [44,45] and organization of physical activity [44] are prevalent among all adolescents, irrespective of weight status. Furthermore, interpersonal barriers such as lack of peer [44] and parental support [46] as well as victimization [44] were reported by all adolescents. Being overweight however, appears to create an additional factor which predisposes this group of adolescents to higher levels of victimization and difficulty in forming peer relationships, all of which are pertinent to physical activity engagement. Similarly, individual level barriers such as competing demands on time [44], low self-efficacy in overcoming transportation barriers [47] and perceived lack of athletic ability relative to peers, although reported by all adolescents, are likely exacerbated by weight status. Consequently, barriers experienced by all adolescents, in addition to those either unique to or exacerbated by obesity, increase overweight and obese adolescents’ psychosocial vulnerability at a critical period of psychosocial development and significantly influence participation in physical activity across settings.

### Psychosocial vulnerability in relation to settings

The school is a key setting for adolescent socialisation [39]. It was prominently featured by nearly half the studies in the review as the setting in which barriers to physical activity occurred. Overweight or obese adolescents were subjected to environmental and interpersonal threats in this context, including bullying [28,30], and social exclusion emanating from a lack of privacy in change rooms [30] or mandated PE uniforms considered revealing of the overweight figure [38]. For some adolescents, these experiences contributed to the cultivation of self-perceptions of inferiority and negative body image during a formative period of identity development and the establishment of relationships, including those with the opposite sex.

Some barriers identified within the school setting were also described in the general context. For instance, at the environmental level, the regulatory environment and inhibitory social norms were identified as major barriers across both settings. Furthermore, it was at the individual level where the majority of barriers were described in the general context. Although more research is required to explicate how barriers are experienced by adolescents in different settings, the lack of differentiation and alignment of individual level barriers with a general context may highlight the pervasiveness of these individual barriers across contexts.

Furthermore, the fact that the barriers to physical activity emerged from studies that focused on topics as victimisation, stigmatisation, mental health and body image is particularly poignant and reflects the potentially pervasive influence of their excessive weight not only in relation to physical activity situations but other aspects of their lives. It also points to a heightened sense of psychosocial vulnerability in overweight and obese adolescents during a formative period of their psychosocial development [7,8]. Adolescents compare themselves to their peers and make judgments about their relative social standing based on how they are treated and what they perceive others to think of them – even if what their peers are thinking is not accurate. Egocentrism is heightened during early adolescence and is characterised by a belief that peers are watching, thinking about and monitoring them [48]. According to Elkind [48], adolescent egocentrism represents a fault in early formal operational thought because adolescents mistakenly believe that others are thinking about them as much as they are thinking about themselves. Nonetheless, this perception is vivid and real and something that adolescents who are overweight or obese appear to experience with heightened intensity particularly in the presence of the opposite sex.

### Gender, ethnicity and socioeconomic status

Six studies included only female participants [10,28,31,33,34,37] giving rise to an over-representation of this gender in the review. Barriers noted as unique to this gender in this review included the perceived “beauty cost” [31] and a lack of resources [34].

The “beauty cost” of messy hair, runny make-up and breaking finger-nails deters at least a sub-group of girls from physical activity which may signal their interest in looking attractive and forming relationships with boys. The findings illuminated in the review also suggest that girls feel hindered by a lack of access to the right exercise equipment. Some research shows that lack of exercise resources was influenced by perceived self-efficacy to overcome barriers [49,50]. One might surmise that a lack of resources would apply to boys as well; however, this did not emerge in our findings because a majority of studies focused on the experience of girls. Studies including both genders are needed to better understand similarities and differences in the barriers to physical activity.

It is difficult to make inferences about the roles of ethnicity and SES in relation to barriers to physical activity. First, most studies included in the review did not focus on physical activity barriers specifically. Second, most studies did not examine differences in experiences of barriers in relation to ethnicity and SES, and for some studies, participants were from the same ethnic or SES group. Given this, it is of interest to note that in one study African-American girls who are overweight did not perceive their weight as an issue and therefore weight-related comments did not motivate them to change their physical activity habits [31]. Furthermore, while only five studies attempted to discuss potential differences between ethnic groups in the discussion section, all but one failed to base these on specific quotations. These discussions concluded that African American girls encountered hurtful situations with strangers while Caucasian girls did not [10] and another expressed that the African American social environment is less negative about obesity [36,37]. However, both studies found similarities between ethnic groups in adolescents’ description of hurtful experiences [10], body and self image dissatisfaction, pertaining to clothing in particular [37]. Differences in cultural norms were also considered and no difference in attitudes was found between black South African adolescents and their western counterparts [33]. Adolescents from low SES backgrounds described barriers including lack of competence [32] and family support [10,32]. Conversely adolescents from relatively high income families reported the least amount of acceptance of overweight individuals [36].

### Limitations

Findings should be interpreted in relation to study quality, which was variable. Few studies explicitly identified their qualitative approach, provided sufficient justification for data collection methods or detail on data analysis. Problems were also noted with researcher reflexivity and strategies used to ensure rigour. Furthermore, a number of studies failed to provide appropriate references for the analysis processes used. Nearly all studies commented on the value of the research, although a number of these failed to identify areas for future research. In addition to concerns regarding study quality, a key limitation of this meta-synthesis was that the majority of included studies, with four exceptions [25-27,34], were not aimed at understanding the barriers to physical activity experienced by adolescents who are overweight. Instead, barriers emerged as themes in studies focusing on other issues including stigmatisation and self-image. Consequently, the findings synthesised lack depth and in some cases are not even supported by quotations. The reporting on ethnicity and SES in primary studies at both the descriptive and synthesised levels was very poor, especially in relation to the review question focusing on barriers to physical activity. The inferences that can be made to address research objectives are thus limited.

### Implications for engaging adolescents in physical activity

The second study objective was to identify implications for adolescent engagement in physical activity. Schools are a powerful context for shaping the lives of young people. Adolescents who are overweight or obese are vulnerable to internalising their experiences with environmental and interpersonal barriers to physical activity at a formative period of their development. Whole-of-school anti-bullying programs that promote the values of respect and fairness will likely be more successful than strategies that perpetuate the stigmatisation and victimisation of adolescents by isolating them or a particular incident out amongst their peers. The quotations suggest that adolescents who carry extra body-weight do not want to be on display; they want to fit in and be a part of the group. School dress codes, particularly for PE clothing, should accommodate larger body sizes so that short or tight fitting clothing can be avoided. Concessions should be made for adolescents to dress for PE in private change rooms or bathroom cubicles rather than in an open changing area. In addition, PE classes should offer activities that are suitable for all athletic and body types, and allow adolescents some choice in the matter. All school staff, but PE staff in particular, would benefit from training that raises their awareness and competencies to address the environmental, interpersonal and individual barriers that overweight and obese adolescents confront in relation to physical activity and other learning situations. This type of training should be extended to parents and other exercise or physical activity providers in community centres and clubs. The paucity of gender specific data limits the recommendations that can be made with respect to gender across settings. Further research is required to address these limitations.

### Implications for future research

Findings from this qualitative synthesis present a first step towards reducing the knowledge gap in understanding the barriers to physical activity for adolescent who are overweight and obese. Given that only four of the 15 studies fit the review question well, further qualitative research is required with a specific focus on understanding the barriers to physical activity. Most studies included in this review offer thin descriptions on some aspects of adolescent’s experiences with barriers to physical activity. Little is known about how adolescents cope in these physical activity situations and how these experiences shape their identity, social relationships and engagement in physical activity in the longer-term. There is a need to focus on potential differences in barriers faced by adolescents of different SES and ethnic groups and the reporting of these in primary studies to facilitate future between group comparisons. The sensitive and stigmatising nature of the topic lends itself to interviewing young adults reflecting back on their adolescent experience. Such an approach would also provide a rich developmental perspective and potentially pinpoint developmental points of particular vulnerability and opportunity. Only when this knowledge gap is addressed will teachers, parents, coaches and others involved in the delivery of exercise and sporting activities, be able to create environments that engage adolescents who are overweight or obese in physical activity.

## Competing interests

The authors declare that they have no competing interests.

## Authors’ contributions

IS carried out the literature searches, did the quality assessment and coding as well as drafted the manuscript. MC conceived the study, completed the quality assessment and contributed to the analysis, writing and editing of the manuscript. TO edited manuscript drafts and provided feedback on the literature search and analysis of codes. All authors read and approved the final manuscript.
